# Mapping the Geographic Distribution of Dimorphic Mycoses Using a US Commercial Insurance Database

**DOI:** 10.1093/ofid/ofae755

**Published:** 2025-10-10

**Authors:** Reid Goodman, Adriana M Rauseo, Samuel L Windham, Julio C Zuniga-Moya, William G Powderly, Andrej Spec, Patrick B Mazi

**Affiliations:** Division of Infectious Diseases, Department of Medicine, Washington University in St Louis School of Medicine, St Louis, Missouri, USA; Division of Infectious Diseases, Department of Medicine, Washington University in St Louis School of Medicine, St Louis, Missouri, USA; Division of Infectious Diseases, Department of Medicine, Kansas University School of Medicine, Kansas City, Kansas, USA; Division of Pulmonary and Critical Care Medicine, Department of Medicine, Kansas University School of Medicine, Kansas City, Kansas, USA; Division of Infectious Diseases, Department of Medicine, Washington University in St Louis School of Medicine, St Louis, Missouri, USA; Division of Infectious Diseases, Department of Medicine, Washington University in St Louis School of Medicine, St Louis, Missouri, USA; Division of Infectious Diseases, Department of Medicine, Washington University in St Louis School of Medicine, St Louis, Missouri, USA; Division of Infectious Diseases, Department of Medicine, Washington University in St Louis School of Medicine, St Louis, Missouri, USA; Division of Pulmonary and Critical Care Medicine, Department of Medicine, Washington University in St Louis School of Medicine, St Louis, Missouri, USA

**Keywords:** blastomycosis, coccidioidomycosis, dimorphic, endemic mycoses, histoplasmosis

## Abstract

**Background:**

The dimorphic mycoses (DMs) histoplasmosis, blastomycosis, and coccidioidomycosis are classically thought to be geographically restricted to specific regions of the United States. The current scientific consensus is that these geographic boundaries are expanding, but the extent of this expansion remains uncertain.

**Methods:**

This study is a retrospective analysis of >21 million (annually) commercially insured individuals within the United States during 2007–2016. Diagnosis of histoplasmosis, coccidioidomycosis, and blastomycosis were identified using *International Classification of Diseases, Ninth/Tenth Revision* codes within the Merative MarketScan database.

**Results:**

There were 37 513 histoplasmosis, 14 987 coccidioidomycosis, and 2207 blastomycosis diagnoses during 2007–2016 within 387 Metropolitan Statistical Areas. DMs were consistently diagnosed outside their historical hyperendemic regions.

**Conclusions:**

New diagnoses of histoplasmosis, coccidioidomycosis, and blastomycosis were routinely made outside their historically defined regions of endemicity. While exposure to hyperendemic regions remains a risk factor for developing a DM infection, clinicians should consider testing any patient presenting with a compatible clinical syndrome, regardless of their geographic history. Increased suspicion of DM infection will lead to fewer missed or delayed diagnoses, thereby improving patient outcomes.

## Teaser

This retrospective analysis of commercially insured individuals within the United States during 2007–2016 showed that new diagnoses of histoplasmosis, coccidioidomycosis, and blastomycosis were routinely made outside their historically defined regions of endemicity.

Blastomycosis, histoplasmosis, and coccidioidomycosis are invasive fungal infections caused by dimorphic fungi of the genera *Blastomyces*, *Histoplasma*, and *Coccidioides*. As environmental pathogens, these fungi were thought to be geographically restricted to specific regions of the United States (US), leading to their designation as “endemic mycoses” (EMs). Historically, *Blastomyces* and *Histoplasma* were confined to the Ohio and Mississippi River valleys and *Coccidioides* was limited to the southwestern US, along the US–Mexico border [1, 2]. The geographic distributions of histoplasmosis and coccidioidomycosis were derived from skin antigen testing of military recruits in the 1950s [1, 2]. The geographic distribution of blastomycosis was estimated using case report and outbreak data [1, 2]. For nearly 70 years, a patient's risk to develop an EM has been predicated on their residence in or travel to a historically endemic region based on these classic studies [3–13]. This rigid, binary risk assessment persists in current medical practice despite EMs being increasingly diagnosed beyond their classical endemicity borders [13–15].

Geographic expansion of EMs was predicted by mathematical modeling [10, 16, 17] and further supported by soil, environmental, and genomic testing [18–22], expert opinion [11, 12, 23–25], public health data [26–28], and large-scale healthcare database analyses [15]. The available evidence supports evolving EM epidemiology, but the actual geographic distribution is not yet definitively confirmed. Due to the uncertainty placed on the term “endemic,” we will avoid using it for the remainder of the manuscript. We will refer to blastomycosis, histoplasmosis, and coccidioidomycosis as dimorphic mycoses (DMs). Regions within classical DM geographic distributions will be described as hyperendemic to recognize the higher incidences in these areas, while also acknowledging that other areas of endemicity exist.

While multiple factors likely contribute to the expanding epidemiology of DMs, anthropogenic climate change has repeatedly been implicated as a major driver [10, 16, 17, 24, 29–34]. DMs maintain environmental reservoirs and do not require a host for survival. Thus, as true environmental pathogens, DMs are fundamentally connected to their local ecosystems and intrinsically linked to climate change. DMs are well suited to flourish amid rising global temperatures, unstable climate variables (eg, precipitation, wind), and the increased frequency of extreme weather events [34].

We recently published modern maps based on DM diagnoses using a cohort of ≥45 million Medicare patients [15]. The approach and interpretability of our findings is limited by patient age—the cohort consisted of patients aged 65 years or older—and our inability to account for travel history and related exposures [35]. While we are not able to fully mitigate these limitations, we aimed to perform a similar analysis using a different cohort, the Merative MarketScan Commercial Database, that is both younger and may have different factors affecting travel behavior.

## METHODS

We used the Merative MarketScan Commercial Claims and Encounters database during 2007–2016. This cohort includes persons, spouses, and dependents covered by employer-sponsored private health insurance in the US. Patients <18 years old were excluded from analysis. Infections were identified using *International Classification of Diseases, Ninth Revision* (*ICD-9*) (histoplasmosis 115.x, coccidioidomycosis 114.x, blastomycosis 116.x) and *International Classification of Diseases, Tenth Revision* (*ICD-10*) diagnostic codes (histoplasmosis B39*, coccidioidomycosis B38*, blastomycosis B40*). To avoid misclassification, DM diagnoses based on radiographic and/or laboratory claims were excluded. The first nondiagnostic claim coded for a DM diagnosis was counted as the incident diagnosis.

The Metropolitan Statistical Area (MSA) designation coded for each enrollee was used for the geographic location of their DM diagnosis. Individuals coded with a non-MSA designation were included in the total number of enrollees, but their data could not be analyzed for mapping purposes. MSAs were defined by the US Office of Management and Budget. There are 387 MSAs, which consist of at least 1 urban area of ≥50 000 inhabitants and surrounding communities that are linked by social and economic factors. Each DM incidence was defined as the number of persons diagnosed divided by the available enrollees for each year and summated as a 10-year total for each MSA.

We attempted to mirror our previous analysis of Centers for Medicare and Medicaid Services (CMS) claims data [15]. Due to the construction and subsequent composition of each cohort, there are notable differences. The CMS cohort includes all persons aged 65 years and older and uses each patient's home address (county) for the location of DM diagnosis. The MarketScan cohort includes the primary policy holder of the private and/or employer health plan and their dependents aged 18 years or older, and DM infections were mapped by MSA rather than county.

We intentionally chose to represent our findings as annual totals, 10-year cumulative totals, and maps of DM incidence by MSA. Due to intrinsic biases in our data, specific incidences are unlikely to accurately quantify real-world DM geographic epidemiology. However, a consistent approach across geographic location allows for comparative incidence to be visualized and permits general geographic trends to emerge.

Incidence rates were mapped for each MSA using R software version 4.1.2 (R Foundation for Statistical Computing, Vienna, Austria). Colorization thresholds for incidence rates for each DM map were optimized for visual discrimination.

This study was approved by Washington University's Institutional Review Board with a waiver of consent as no individual patient protected health information was collected.

## RESULTS

Among commercially insured cases in our MarketScan cohort, there were 37 513 cases of histoplasmosis, 14 987 cases of coccidioidomycosis, and 2207 cases of blastomycosis between 2007 and 2016 (Table 1). Histoplasmosis was the most common DM annually and had the highest 10-year cumulative total. There were fewer diagnoses annually with a lower 10-year cumulative total for each DM compared to the Medicare fee-for-service cohort [15]. However, the distribution of DM diagnoses was proportionally similar in both datasets (Supplementary Table 1).

**Table 1.** Number of Annual New Diagnoses of the Dimorphic Mycoses Among MarketScan Enrollees and Their Adult Dependents

Year	No. of MarketScan Enrollees	Annual No. of Diagnoses			Annual Incidence per 1 000 000 Enrollees		
		Blastomycosis	Coccidioidomycosis	Histoplasmosis	Blastomycosis	Coccidioidomycosis	Histoplasmosis
2007	25 566 448	245	1447	4428	10	57	173
2008	34 701 068	297	1554	4833	9	45	139
2009	34 813 028	281	1604	4291	8	46	123
2010	33 295 729	259	1895	3623	8	57	109
2011	38 124 895	256	2274	4264	7	60	112
2012	38 855 425	239	1978	4058	6	51	104
2013	32 019 123	193	1438	3431	6	45	107
2014	35 006 531	195	1336	4093	6	38	117
2015	21 465 717	118	673	2165	5	31	101
2016	21 312 276	124	788	1967	6	37	92
10-y total	315 160 240	2207	14 987	37 153	7	48	118

**Figure 1.**
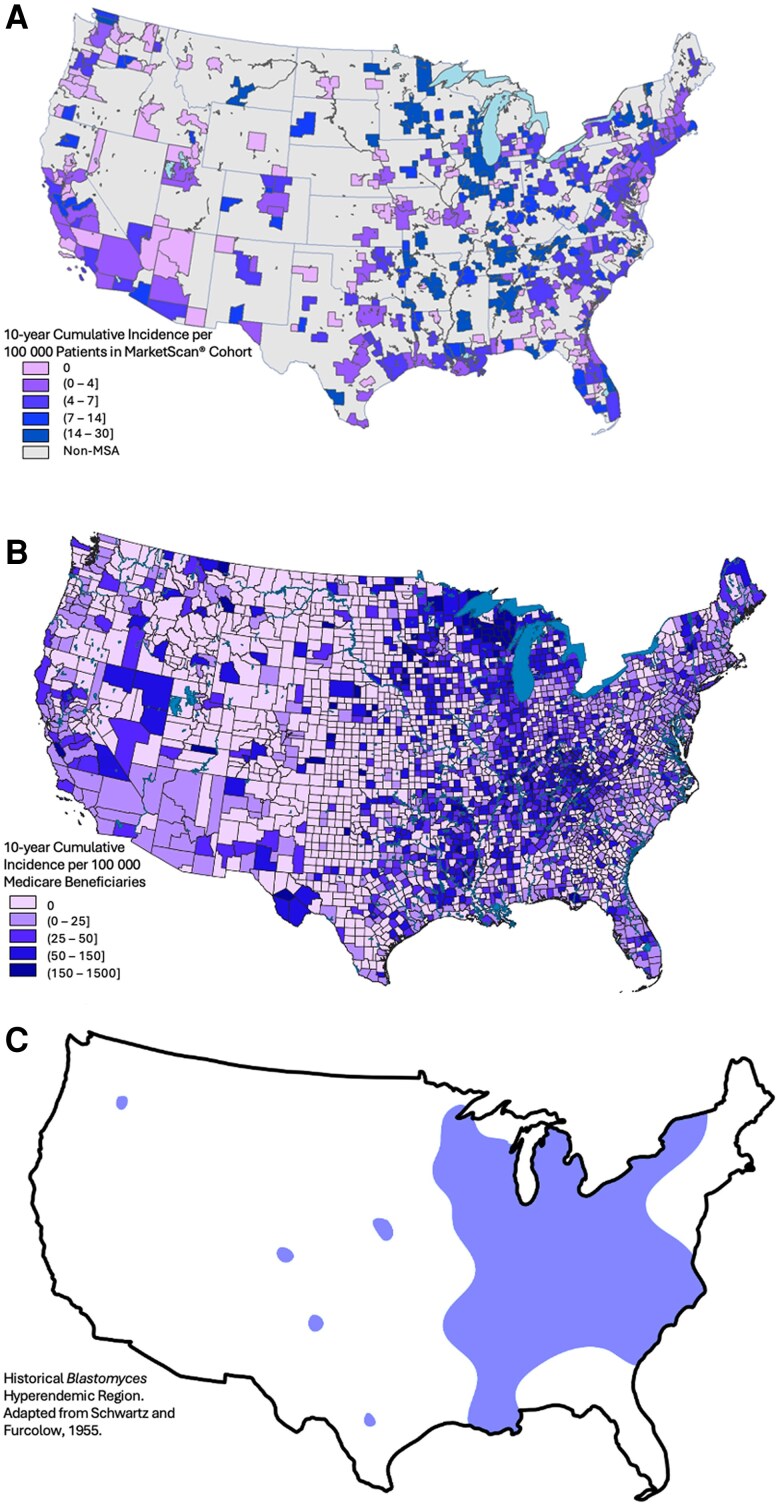
Geographic distribution of blastomycosis by Metropolitan Statistical Area (MSA) utilizing a MarketScan cohort (*A*); United States county utilizing a Medicare fee-for-service beneficiary cohort (*B*); and historical hyperendemic region adapted from Schwartz and Furcolow, 1955 [1] (*C*).

**Figure 2.**
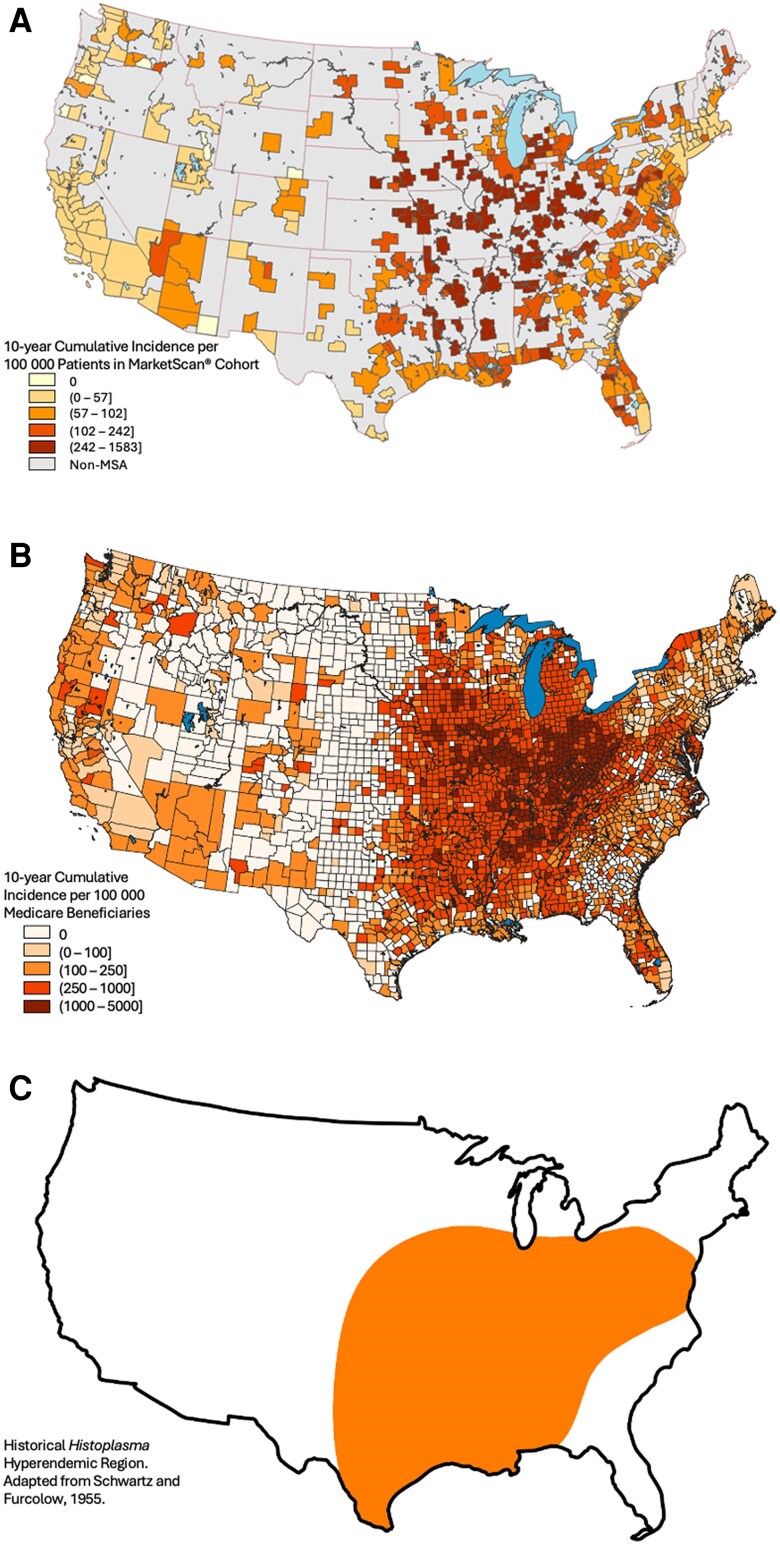
Geographic distribution of histoplasmosis by Metropolitan Statistical Area (MSA) utilizing a MarketScan cohort (*A*); United States county utilizing a Medicare fee-for-service beneficiary cohort (*B*); and historical hyperendemic region adapted from Schwartz and Furcolow, 1955 [1] (*C*).

**Figure 3.**
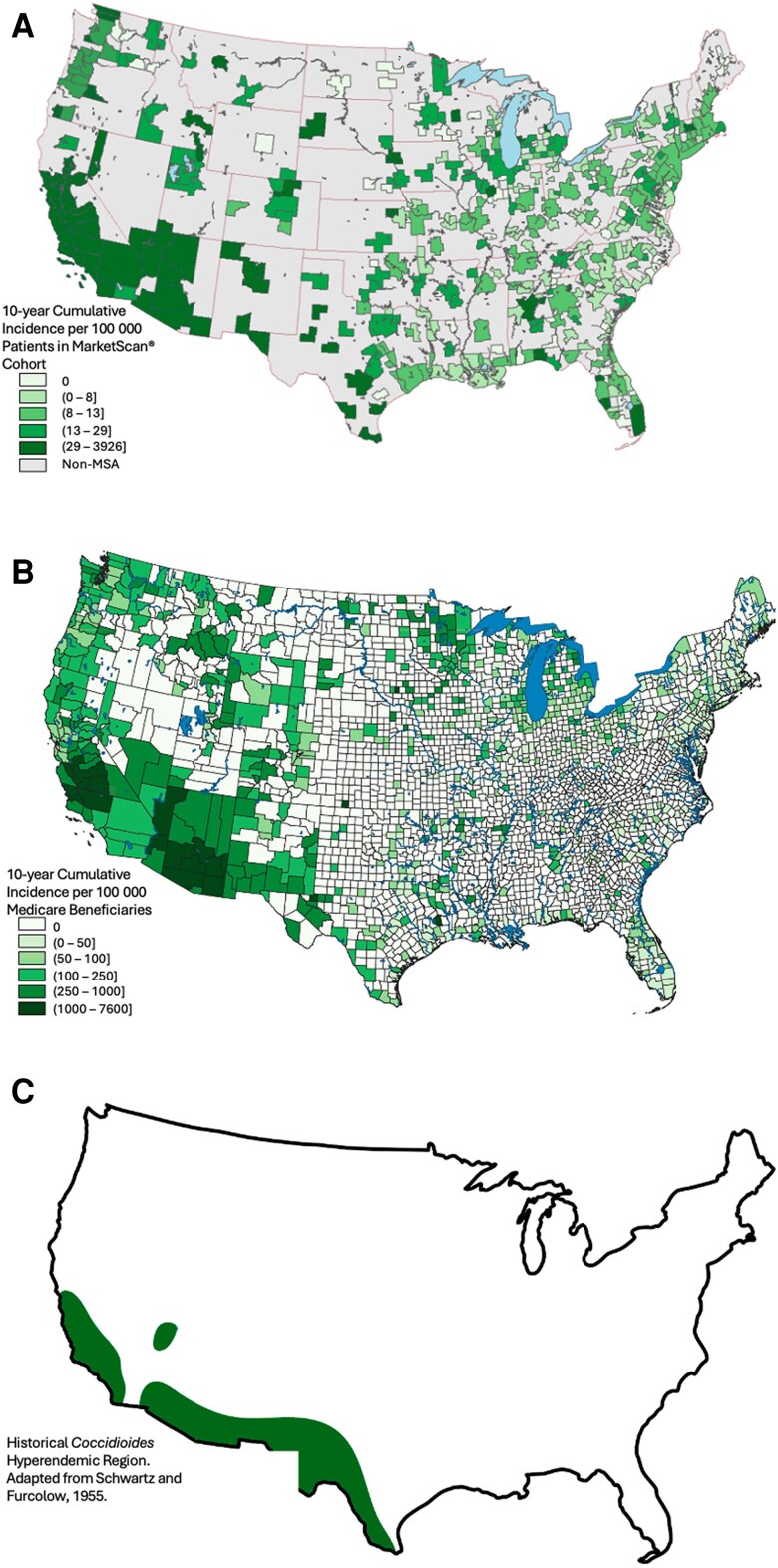
Geographic distribution of coccidioidomycosis by Metropolitan Statistical Area (MSA) utilizing a MarketScan cohort (*A*); United States county utilizing a Medicare fee-for-service beneficiary cohort (*B*); and historical hyperendemic region adapted from Schwartz and Furcolow, 1955 [1] (*C*).

Maps showing the geographic distribution of blastomycosis, histoplasmosis, and coccidioidomycosis diagnoses are presented in Figures 1*A*–3*A*. Maps based on our Medicare cohort analysis (Figures 1*B*–3*B*) and maps of historical DM geographic distributions (Figures 1*C*–3*C*) are included for visual comparison.

## DISCUSSION

The maps defining the geographic distribution of blastomycosis, histoplasmosis, and coccidioidomycosis produced by this study reconfirm historical areas of hyperendemicity and also provide additional support for the scientific consensus that the geographic epidemiology of these fungal pathogens has evolved. A trend toward expansion of DM geographic distributions was identified in our data. These findings mirror the observations from our analysis of the Medicare cohort. Further, the geographic expansion of DM diagnoses identified in both MarketScan and Medicare analyses correlates with the predictions from climate models and expert opinion. There are fewer DM diagnoses in the MarketScan cohort compared to our Medicare cohort [15]. This difference is likely due to the risk profiles of each cohort and reflects a younger—presumably with fewer medical comorbidities—population with lower probability of symptomatic infection.

Histoplasmosis had the widest distribution and area, with the highest incidence expanded northward toward the Great Lakes, southward along the Gulf Coast, and along the eastern US coastline. This observed trend was similar to the histoplasmosis geographic expansion predicted by Maiga et al using a histoplasmosis soil suitability model [10]. In our analysis, coccidioidomycosis remained hyperendemic to the southwestern US, had geographic expansion northward along the US west coast, and extended further along the southern Texas border and northward through the Rocky Mountains and Northern Plains to the US–Canadian border. This expansion pattern is similar to the expanded *Coccidioides* habit predicted by climate modeling from Gorris et al [17, 36]. There are minimal historical or contemporary data predicting the geographic distribution of blastomycosis. Both our MarketScan and Medicare datasets suggest blastomycosis diagnoses outside historical hyperendemic regions. A general trend toward geographic expansion of blastomycosis is consistent with observations from histoplasmosis and coccidioidomycosis; there is no similar predictive modeling for comparison.

The correlation of our administrative data with predictive modeling is not surprising. When investigated, DMs are consistently found to exist in locations far beyond their expected ranges. *Histoplasma* was recently discovered in penguin excreta and soil in Antarctica, meaning the fungus has now been found on all 7 continents [37]. *Coccidioides* was not considered endemic to Washington state (>1000 miles north of its historical endemic region) until a series of 3 locally acquired infections were published [18, 22]. Moreover, an investigation of coccidioidomycosis cases in Missouri found that 26% of the cases were unrelated to travel to an historically endemic region [27]. This percentage is potentially higher as another quarter of cases had unknown travel histories [27]. Previously thought to be relatively rare in New England, the endemic burden of blastomycosis was recently found to be greater than historically appreciated [38].

Despite the preponderance of evidence signaling an evolving DM epidemiology [5–8, 10, 11, 15–19, 22, 24, 26–38], the current approach to DM diagnoses remains hyperfocused on a patient's exposure to classically hyperendemic areas. Ignoring the data and subsequent failure to consider potential DM diagnoses results in patient harm. Half of DM patients experience delayed diagnoses of weeks to months and 80% experience missed opportunities for diagnosis [4, 14, 39, 40]. Delayed/missed DM diagnoses are associated with increased healthcare costs, inappropriate antibiotic use, unnecessary invasive procedures, increased morbidity, and higher mortality [40–43].

To avoid missed or delayed diagnoses, DMs should be considered in patients with a compatible clinical syndrome regardless of exposure to a classically hyperendemic region [25]. Exposure to a hyperendemic region remains an important diagnostic clue and should not be disregarded. However, given the uncertainty of DM geographic distribution, a patient's lack of exposure to a historically hyperendemic region is not sufficient to rule out a DM diagnosis.

We believe this study provides meaningful evidence in support of expanded DM geographic distributions. However, it does not specifically confirm new areas of endemicity/hyperendemicity, or environmental variables associated with DM geographic expansion. Moreover, this study should be interpreted with the following limitations: First, analysis of administrative data has intrinsic biases, including misclassification bias and sampling errors. These biases certainly affect our data, though the size of the cohort makes obscuring general trends unlikely. Second, our analysis includes patients diagnosed with presumed ocular histoplasmosis. Third, the dataset does not represent patients with noncommercial health insurance or patients without insurance. The authors recognize the significance and underrepresentation of this population; however, this limitation is unlikely to affect identification of general trends due to the uniformity of this exclusion across geographic locations. Fourth, our MSA-based mapping excludes patients residing in rural areas. Rural areas have previously been associated with higher incidence of histoplasmosis than metropolitan areas [44]. Mapping DM diagnoses within MarketScan MSAs likely caused artificial concentration of cases around larger regional healthcare centers. This limitation predisposes our findings toward geographic restriction due to referral bias. Fifth, our analysis is based on claims data and does not account for travel-related exposures. However, there is general agreement in mapping trends between the MarketScan and Medicare cohorts representing >100 million patients. The large number of patients included will somewhat mitigate the effect of individual travel. Regardless of the location of exposure, these maps still demonstrate that patients were diagnosed outside the historical regions of endemicity.

**Figure 4.**
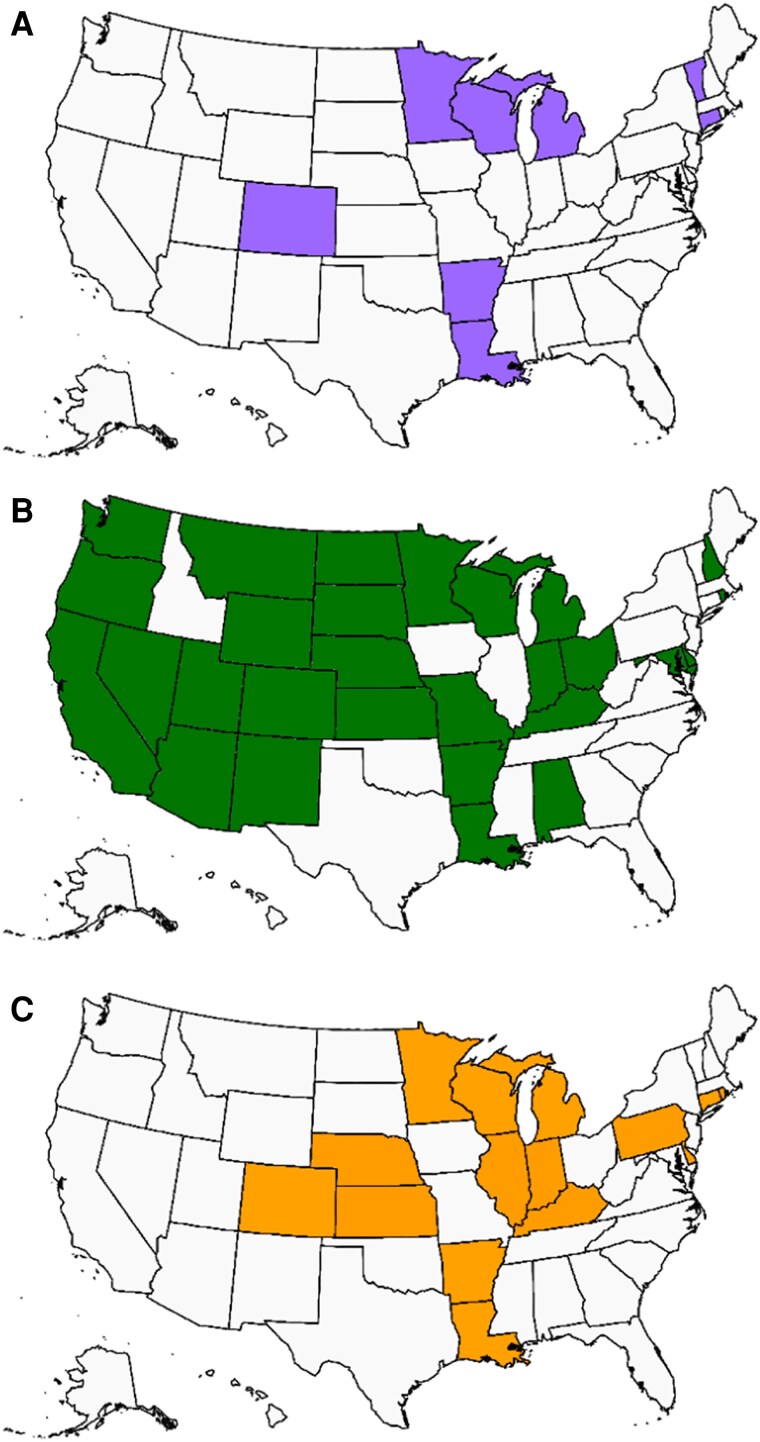
States with mandatory reporting for the dimorphic mycoses. Blastomycosis is reportable in 8 states (*A*); coccidioidomycosis is reportable in 29 states (*B*); and histoplasmosis is reportable in 15 states (*C*).

Our repeated analyses reporting similar observations of DM epidemiology support the scientific consensus that the geographic distribution of these pathogens has expanded, but definitive confirmation remains elusive. Efforts to conclusively remap DM epidemiology face several challenges. Coccidioidomycosis is a reportable disease in only 29 states, histoplasmosis in 15 states, and blastomycosis in 8 states, limiting the potential contributions from public health entities (Figure 4*A*–*C*). Repeating the historical skin antigen studies is prohibitive due to potential ethical issues involving a vulnerable population and lack of available testing reagents. Environmental sample testing would be highly informative but is cost prohibitive. This issue is exemplified by a recent study of soil samples from Washington state investigating environmental *Coccidioides* following a confirmed outbreak. Despite investigating in the environment around a confirmed coccidioidomycosis outbreak, 75% of environmental samples collected within 100 meters of that location were not positive for *Coccidioides* [18]. Therefore, a multifaceted approach will be required to better delineate DM geographic distributions and identify novel risk factors for patients to develop DMs.

Climate change and other anthropogenic environmental impacts (eg, deforestation, urbanization, desertification) contribute to the emergence of new pathogens, the return of old pathogens, and the spread of infectious diseases [24]. Climate changes associated with rising global temperature favor the geographic expansion of infectious diseases, a pattern observed in our maps of EM geographic distribution [34]. Predicting where this expansion is likely to occur in the future and how it will affect the epidemiology of various infectious diseases is paramount to preparing public health and medical infrastructure in advance of outbreaks and epidemics. The scientific community has predicted the impact of climate change in general and as it relates to infectious diseases for decades. The problem is well-stated; it is time to devote more resources to develop solutions. Better understanding of the relationships between climate change and infectious diseases is needed to predict potential outbreaks and mitigate the effects on humanity.

## CONCLUSIONS

Historical endemicity maps underestimate the current geographic boundaries of histoplasmosis, blastomycosis, and coccidiomycosis within the US. Patients within historically high-risk areas may have a higher pretest probability of testing positive; however, dimorphic fungal infections should be considered in any patient with compatible clinical syndrome regardless of geographic exposure. Due to the close association with climate change, additional research is needed to provide clinicians further insights to aid in the diagnosis of these infections.

## Supplementary Material

ofae755_Supplementary_Data

## Supplementary Data

Supplementary materials are available at *Open Forum Infectious Diseases* online. Consisting of data provided by the authors to benefit the reader, the posted materials are not copyedited and are the sole responsibility of the authors, so questions or comments should be addressed to the corresponding author.

## Notes

***Financial support*.** This work was supported by a Washington University Institute of Clinical and Translational Sciences grant from the National Center for Advancing Translational Sciences of the National Institutes of Health (grant number UL1 TR002345) and by Mayne Pharma.
