# Supplementary material for: Mapping the Geographic Distribution of Dimorphic Mycoses Using a US Commercial Insurance Database
Source: Open Forum Infect Dis. 2025 Oct 10;12(10):ofae755. doi: 10.1093/ofid/ofae755 (PMC12548790; doi:10.1093/ofid/ofae755)

Supplementary Table 1: Comparison of the Number of Dimorphic Mycoses Diagnoses Between the MarketScan and Medicare Fee-for-Service Cohorts


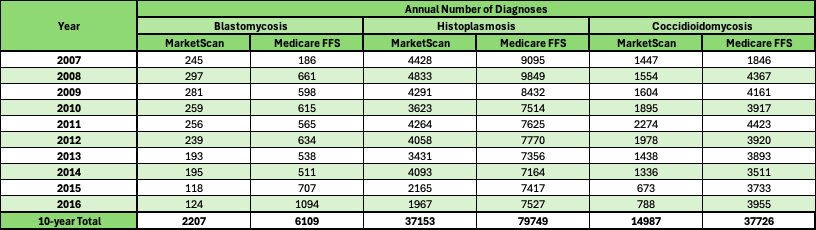

Supplement: ofae755_Supplementary_Data [file ofae755_supplementary_data.docx]
